# The Effect of Diet Based on Legume Seeds and Rapeseed Meal on Pig Performance and Meat Quality

**DOI:** 10.3390/ani10061084

**Published:** 2020-06-23

**Authors:** Anna Zmudzińska, Bartosz Bigorowski, Mirosław Banaszak, Aleksandra Roślewska, Marek Adamski, Marcin Hejdysz

**Affiliations:** 1 Department of Animal Breeding, Faculty of Animal Breeding and Biology, UTP University of Science and Technology in Bydgoszcz, Mazowiecka 28, 85-084 Bydgoszcz, Poland; zmudzinska@utp.edu.pl (A.Z.); bartosz.bigorowski@utp.edu.pl (B.B.); miroslaw.banaszak@utp.edu.pl (M.B.); adamski@utp.edu.pl (M.A.); 2 Department of Animal Physiology, Physiotherapy and Nutrition, Faculty of Animal Breeding and Biology, UTP University of Science and Technology in Bydgoszcz, Mazowiecka 28, 85-084 Bydgoszcz, Poland; roslewska@utp.edu.pl; 3 Department of Animal Breeding and Product Quality Assessment, UP Poznań University of Life Sciences, Wołyńska 33, 60-637 Poznań, Poland

**Keywords:** swine, quality of meat, protein, daily weight gain, fattening period

## Abstract

**Simple Summary:**

Pig diets are primarily composed of cereal-based ingredients and contain soybean meal as the main protein source. A growing trend for increased protein requirements has been noted in animal nutrition. The price of extracted soybean meal fluctuates significantly, hence it is worth replacing it with mixtures based on domestic sources of protein. One of the factors that have a direct impact on the quality of meat is animal nutrition. The aim of this work was to assess the impact of a total dietary replacement of extracted soybean meal (SBM) on the body weight gain and pork quality. The fattening pigs, which were offered diets based on SBM, had similar raw meat parameters as the pigs which were fed diets based on legumes and rapeseed as protein sources.

**Abstract:**

The aim of this work was to assess the impact of a total dietary replacement of extracted soybean meal (SBM) on body weight gain and pork quality. DanBred hybrid piglets were divided into four groups of 10 piglets each. Groups I (males) and II (females) were the control groups and fed a standardized SBM-based complete feed. The experimental groups III (males) and IV (females) were offered a diet in which the SBM was replaced with extracted rapeseed meal (RSM) and legume plants (pea and yellow lupin). After 83 days of fattening, the animals were slaughtered. Based on the collected data, the daily weight gain (DWG), feed intake (FI), and feed conversion ratio (FCR) were calculated. In addition, longissimus dorsi muscle was subjected to physicochemical analyses, including the basic chemical composition. All the analyses were performed in accordance with the applicable methodologies. As a result of this experiment, no interactions were found between the experimental factors (sex and diet). The replacement of SMB by legumes and RSM resulted in a significant reduction in the final body weight of growing–finishing pigs. Additionally, daily body weight gain was reduced between 35–83 days, and through the whole fattening period (0–83 days). Most pork meat quality parameters were not affected by the type of mix feed and sex (*p* > 0.05). The fattening pigs that were fed legume seeds and RSM had significantly reduced fatness (*p* > 0.05) compared to the control pigs. In males, there was also a significantly lower pH_45_ (*p* < 0.05). It can be concluded that feeding pigs with diets containing legume plants and extracted rapeseed meal does not affect the pork meat quality, but it may worsen the body weight gain.

## 1. Introduction

The animal diet is an environmental factor which directly influences meat quality. According to Aaslyng and Hviid [1], meat quality is characterized by such parameters as: pH, color, taste, succulence, and tenderness.

The meat chemical composition (crude protein percentage, fat, carbohydrates, and water content) is one of the major features that translates to meat value. Pig diets are primarily composed of cereal-based ingredients and contain soybean meal (SBM) as the main protein source. It should also be noted that a demand for protein in livestock nutrition has increased. The domestic production of legume plants, including pea, lupin (white, yellow, narrow-leaved), faba bean, and soy, covers only 30% of the protein requirement. The remaining 70% is sourced from imported SBM [2]. The import of genetically modified soy has increased and market prices have significantly fluctuated. Moreover, the imported non-genetically modified SBM is considerably more expensive compared to a GMO SBM. Therefore, research on the possibilities of replacing GMO soybean meal in the nutrition of monogastric animals, particularly pigs, gallinaceous poultry, and waterfowl, has been performed [2,3,4,5,6,7]. In the past, the use of legumes, mainly lupin in monogastric animal nutrition, was limited due to high alkaloid contents that negatively affect growth, feed intake, and nutrient utilization [8]. During recent decades, however, plant breeders have succeeded in developing lupin cultivars with very low alkaloid contents [9,10], which improved their nutritional value for monogastric animals. However, legumes and rapeseed meal contain antinutrients, such as phosphorus in its phytic form and raffinose family oligosaccharides [11,12]. It has been reported that phytic P depresses protein/amino acid (AA) utilization, due to the formation of complexes, which alter protein structure, and in turn decrease protein solubility, enzymatic activity, and proteolytic digestibility [13,14]. Meanwhile, the high content of raffinose type oligosaccharides [RTO] in the alimentary tract may result in fluid retention and an increased flow rate of digesta, which adversely affects the utilization and absorption of nutrients. These compounds are not broken down in the small intestine but ferment in the hind gut, resulting in flatulence and the disruption of digestion [15]. Legumes and rapeseed meal contain higher contents of these antinutritional factors than SBM, and therefore may negatively influence pig performance.

Meat quality has been studied in pigs that were offered complete feeds with a share of legume seeds and rapeseed meal [RSM]. Most often, the authors applied a partial replacement of SBM with yellow lupin, narrow-leaved lupin, pea, or RSM in experimental diets [16,17], whereas the control diets contained SBM. In those studies, the feed components did not negatively affect pork meat quality and the evaluated parameters did not differ from the meat of fattening pigs that were fed with SBM. Nevertheless, it seems justified to study a complete replacement of SBM with legumes and RSM in complete swine feeds and its effect on pork meat quality.

Here, the hypothesis was that a complete replacement of SBM with legumes and RSM would not adversely affect the body weight gain and meat quality of pigs.

The aim was to determine the effect of a complete replacement of SBM with legumes and RSM in pigs’ diets on body weight gain and meat quality in both sexes.

## 2. Materials and Methods

No individual approval of an ethics committee was required for this study. The experimental animals were slaughtered in a registered meat processing plant. The studies were part of a routine production cycle in the swine production sector. The major research goal was to analyze the carcass and meat quality (Directive No. 2010/63/EU).

### 2.1. Legume Seeds

Legume seeds, peas (*Pisum sativum*, Tarchalska variety) and yellow lupins (*Lupinus luteus*, Mister variety), were obtained from a plant breeding station (Wiatrowo, Poland). Before the experimental diet preparation, the above components were ground using a hammer mill, RG11 model (Zuptor, Gostyń, Poland) with a screen size of 2.0 mm. The chemical composition of the studied protein components is presented in Table 1.

### 2.2. Animals and Diets

DanBred hybrid pigs were imported from Denmark through the company PIGRO. The piglets had an average body weight of 30 kg. The animals were divided into four groups and kept in identical animal enclosures, 10 piglets/per box (one box = one group), in identical environmental conditions, on a deep litter.

The experimental groups were diversified by sex (males and females) and the diets. Diets varied in terms of protein components. The animals in groups I and II were fed complete feeds based on SBM, whereas in groups III and IV, the complete feeds contained legume seeds and RSM. The housing density was 1.0 m^2^ per pig. The pigs were given access to complete pellets (automatic feeders) and water (nipple drinkers). The body mass was measured at three time points during the fattening period: at arrival to the farm (1 day), with the change of the grower feed type (25 days), and at slaughter (83 days). For each group, the feed intake (FI) was registered and feed conversion ratio (FCR) was calculated. The composition of mixed feed (Table 2) was prepared according to the nutrient requirements and feed nutritive value stipulated in the applicable standards for pig nutrition [18]. At the end of the fattening period, the animals with a weight of 113.6–120.2 kg were slaughtered in the meat processing plant. Following the pre-slaughter transport, the animals were allowed to rest with free access to water. An electric stunning method was applied to the pigs.

### 2.3. Meat Quality

The carcass weight was determined with a pig carcass weighing scale 45 min *post mortem*, in the meat processing plant. The carcass meatiness was estimated using an Ultra Form device. In the same location, an initial measurement of the longissimus dorsi muscle acidity (pH_45_) was taken and the second measurement was taken 48 h after slaughter (pH_48_) using a CX-701 pH-meter with a dagger electrode (Elmetron). The half carcasses were cold-stored at 4 °C in the slaughterhouse. Twenty-four hours after slaughter, the fat thickness was measured in a right half carcass using a caliper [19]. To evaluate pork meat quality, the samples were excised from longissimus dorsi muscle. The other analyses, like meat color, water holding capacity, thermal drip, meat hydration, and meat chemical composition, were performed 48 h after slaughter. The meat color was determined using a colorimeter (Konica Minolta, model CR400, Tokyo, Japan). The standardization of the device was completed using a white calibration plate (no. 21033065) and the surface color was estimated according to the CIE L*a*b* system (L*—lightness, a*—redness, b*—yellowness values) [20]. Water holding capacity was carried out according to the Honikel method [21]. Thermal drip was determined according to Walczak [22]. Meat hydration was estimated using a modified method of Grau and Hamm [23].

Moreover, the analysis of the meat chemical composition was performed. The content of crude protein, intramuscular fat and water were estimated according to applicable standards [PN-A-82109:2010] [24]. Meat analysis was conducted using a food scan device (FOSS) based on artificial neural network (ANN) calibration and near-infrared red (NIR) transmission spectroscopy.

### 2.4. Analytical Methods

Chemical analysis was performed for the pea seeds that were used as ingredients in the complete feed mixes in this study. The seeds were ground using a 0.5 mm mesh filter. Crude protein (CP) was determined according to the Association of Official Analytical Chemists method 976.05 (AOAC 2007, [25]). The levels of neutral detergent fiber (NDF), acid detergent fiber (ADF), and residual ash were determined according to the AOAC 942.05 and 973.18 methods. Starch content in pea seeds was determined using a diagnostic assay kit for agricultural industries (Megazyme International; AOAC, 2005: Method 996.11, Dublin, Ireland) based on the use of thermostable α-amylase and amyloglucosidase. The AA content was determined using an AAA-400 Automatic Amino Acid Analyzer (INGOS s.r.o., Prague, Czech Republic) and postcolumn derivatization with ninhydrin reagent (procedure 994.12; AOAC, [25]). The tannin content was analyzed according to the vanillin–sulphuric acid method of Kuhla and Ebmeier, [26] with catechine as a standard. The raffinose family oligosaccharides (RFO) were extracted and analyzed by high-resolution gas chromatography, according to Zalewski et al. [27]. The phytate content was determined according to the method of Haug and Lantzsch [28]. Briefly, samples (0.025 g) were extracted in hydrochloric acid (5 mL; 0.2 M) for 3 h, and subsequently iron ammonium sulfate (1 mL) was added to the 0.5 mL of centrifuged extract (5 min; 3000 rpm). Then, the extract was heated (30 min at 100 °C) and centrifuged (5 min; 3000 rpm). Bipyridine solution (1.5 mL) was added to the supernatant (1 mL). Absorbance was determined using a media spectrophotometer (Marce Lamidey S.A., Châtillon, France) at 519 nm wavelength. Lupin alkaloids were extracted from lupin seed flour with trichloroacetic acid and methylene chloride (Sigma-Aldrich, Munich, Germany). The determination of alkaloids was carried out via gas chromatography (GC) (Shimadzu GC17A, Kyoto, Japan) using a capillary column (Phenomenex, Torrance, CA, USA).

### 2.5. Statistical Analysis

All data were explored prior to statistical analysis to discard any possible outliers. Analyses were performed using the appropriate procedures on SAS Software (distribution analyses; outliers were defined as the observations in which distance to the estimated location was exceeded by three times the standard deviation). The obtained results were subjected to two-factorial analysis of variance. Differences were considered significant at *p* ≤ 0.05 and significant differences between means were identified by Duncan’s test.

## 3. Results

### 3.1. Chemical Composition of Legumes

The protein ingredients were characterized by a high variability in terms of chemical composition. The highest CP content was found in SBM content, with 465 g/kg dry matter (DM) whereas the lowest CP content was found in pea seeds, at 213 g/kg DM. High differences were also found for ADF and NDF. The highest NDF concentration was observed in yellow lupin seeds (245 g/kg DM), whereas the lowest ADF levels were found in SBM (79 g/kg DM) and peas (92 g/kg DM). Starch content was determined at a level of 578 g/kg DM in the pea seeds. The content of alkaloids was determined at a level of 0.0011 g/kg DM in the yellow lupin seeds. Moreover, the yellow lupin seeds contained higher levels of raffinose family oligosaccharides (134.2 g/kg DM) and raffinose (10.2 g/kg DM), whereas the lowest content of those compounds was found in the rapeseed meal.

### 3.2. Fatteners Performance

No mortality was found in any group and no veterinary service was required. In this study, no interaction between the experimental factors was observed (sex, diet) (Table 3). No significant differences in body weight were observed among the pigs that were fed a diet based on legume plants, seeds, and rapeseed meal, across the whole fattening period (83 days). The analysis of the whole fattening period showed a significant decrease in the pigs’ body weight due to replacing SBM with the examined plant protein sources. In the first period (grower) of the experiment (0–35 days), there was no effect of the complete feed mix on daily weight gain (*p* < 0.05). Considering the second period (finisher—36–83 days) and the whole fattening period (1–83 days), a significant decline in daily weight gain was recorded as a result of the inclusion of legume plants, seeds, and RSM to the complete feed mixes (*p* < 0.05). No significant impact of pig sex on the evaluated performance parameters (*p* < 0.05) was found here. Those parameters did not differ between sexes. However, a numerical difference was found for FI between the groups with different diets. In groups with the diets based on SBM (groups I and II), the average FI and FCR were as follows: FI for 0–35 days—1.75 kg, 36–83 days—3.14 kg, 0–83 days—4.90 kg, and FCR for 0–35 days—2.59 kg/kg, 36–83 days—2.77 kg/kg, 0–83 days—2.70 kg/kg. Meanwhile, in pigs that were fed diets containing legume plants and RSM (groups III and IV), these parameters were as follows: FI for 0–35 days—1.62 kg, 36–83 days—3.38 kg, 0–83 days—5.01 kg, and FCR for 0–35 days—2.48 kg/kg, 36–83 days—3.22 kg/kg, 0–83 days—2.93 kg/kg.

### 3.3. Meat Traits

Regarding meat quality, no interaction between the experimental factors was found in this study. For the back fat thickness recorded from five measurements, significant differences were found between the animals that were fed with the SBM diet (2.26 cm) vs. the RSM diet enriched with legume plants (1.99 cm) (Table 4). Regarding the acidification of the muscle tissue 45 min *post mortem*, a significant difference was observed between the sexes. In females, the pH_45_ was 6.10, whereas in males it was 6.07. The next examination of pH_48_ *post mortem* gave the same result, 4.35, for gilts and barrows (Table 5). Apart from the abovementioned indices, neither the sex nor the diet affected the other pork meat quality parameters determining the technological suitability of the meat. Carcass meatiness was found to be similar in each animal group in this study. Additionally, similar results were observed regarding the water holding capacity, thermal drip, drip loss, and meat colour in the experimental and control groups. The chemical composition of the pork meat was analyzed in this study (Table 6). The results were found to be similar in all the groups. Protein content in the longissimus dorsi was higher but not statistically significant in the animals that were fed a diet with RSM and legume plants (23.82%) compared to the fattening pigs that were fed a diet based on SBM (23.67%).

## 4. Discussion

### 4.1. Chemical Composition of Legumes

In this study, the cultivars of legume plants and soybean meal were characterized by a similar concentration of crude protein (CP), amino acids (AA), and starch as in the studies of Nalle [29] and Abdulla et al. [30]. Similar to our study, Hanczakowska et al. [31] used the same lupin variety (Mister), though it contained a higher concentration of CP. Hanczakowska et al. [31] recorded a lower concentration of NDF in the peas and yellow lupin compared to the varieties used in our study. Nevertheless, those values fitted the range for varieties presented by Nalle [29]. A similar content of RFO was found by Gdala and Buraczewska [32] in the seeds of yellow lupin, but, in the case of narrow-leaved lupins, the content was approximately 15 g/kg DM lower than that determined in the current study. In the case of peas, the level of RFO was approximately 8 g/kg DM lower compared to a study presented by Hejdysz et al. [33]. This difference could be due to the genetics of the cultivars, agronomic management, growing location, and climatic conditions [34,35].

### 4.2. Fatteners Performance and Meat Quality

In the first fattening phase, the pigs consumed 1.75 kg of feed daily in the control group, and 1.62 kg in the experimental group. Lower consumption in the experimental group could result from a lower tastiness of the feed mix, in which the legume seeds (yellow lupin, pea) and RSM were included, or due to the different post-prandial glycaemic response of legume seed-based diets registered in some studies conducted [36,37,38]. According to Hanczakowska and Ksiezak [39], the lupin alkaloids may negatively affect the taste of feed, and therefore the pigs, especially the young ones, are less eager to eat it. Sobotka et al. [40] showed that glucosinolates in rapeseed may also negatively affect the tastiness of feed. On the contrary, in the second fattening phase, the experimental pigs consumed more feed than the control ones. This could be a consequence of a lower feed digestibility. Considering the chemical composition of legume seeds, one should pay attention to a fact that the levels of ADF and NDF, as well as antinutritional factors (including phytic P and RFO), are quite high, which directly influences feed conversion ratio and its digestibility [40]. In another study, a partial replacement of SBM with yellow lupin was proposed in a diet of (landrace × Yorkshire) × Duroc hybrids [41]. The varied percentage content of yellow lupin was applied in animal diets. Namely, it was 7.5% in group II, 15% in group III, whereas the control group (I) was fed with complete feed mixes containing SBM. In this study, the pigs from groups I and II had a similar weight gain in the first fattening period, but in group III, where the content of yellow lupin in the diet was higher, the pigs had a lower weight gain. On the contrary, the pigs from group III had increased weight gain compared to the control group in the finishing fattening period. In terms of daily feed intake, the results recorded by those authors are similar to our results. Summarizing, the pigs in groups I and II had significantly higher body weight gain compared to the animals in groups III and IV, across the whole rearing period (83 days).

Sobotka [42] replaced the SBM diet with a mix of peas and SRM. The content of peas varied between 8–15%, whereas the content of SRM was at a level of 10–15%. In the first fattening phase, the experimental group had a lower daily weight gain, but no differences were found in the second fattening phase. Body weight gain in the experimental groups was lower by about 4% through the whole fattening period, which the author explained by a decreased digestibility. Additionally, in their further study, Sobotka et al. [40] partially (50%) replaced SBM in a feed mix. The protein source in their experimental diet was RSM “00”. The pigs were fattened after they had reached a weight of 65 kg and up to 115 kg. That period corresponded to the second fattening phase of this study. The daily weight gain was lower than observed here (different genotypes), however it was also better in the group fed a SBM diet. The FCR in that study was in the range of 3.14 kg/kg (SBM group) and 3.29 kg/kg (SBM + RSM group), which the authors explained by the effect of glucosinolates in the rapeseed. Okrouhlá et al. [43] observed similar results of fattening as here. They studied the effect of replacing SBM with RSM on fattening outcome in DanBred × (CLW (Czech Large White) × CL (Czech Landrace)) pigs. They observed lower daily feed intake than here (control—2.78 kg: experimental group—2.56 kg). Daily weight gain exceeded 1000 g independent of the diet. The feed conversion ratio was slightly lower in the cited study. Plazak et al. [44] studied body weight gain in PLW (Polish Large White) x PL (Polish Landrace) hybrids that were fed a complete feed mix with peas, potato protein and fish meal in the experimental group, and SBM in the control group. The fattening period lasted 98 days until the pigs gained ca. 100 kg of body weight. Average daily feed intake was about 2 kg (group I—2.14 kg; group II—2.23 kg). The feed conversion ratio was 2.87 kg in group I and 2.90 kg in group II. The experimental group had a 3.2% higher average body weight gain than the control group (group I—745 g, group II—769 g), however that difference was not statistically significant. The authors explained that a better tastiness of the feed and fiber content in the control diet determined a higher feed intake and higher body weight gain. Comparing those results to our study, a lower feed intake and lower body weight gain was observed by Plazak et al. [44] in their PLW × PL hybrids than here. The feed conversion ratio was similar.

Here, the average back fat thickness from five measurements was significantly greater in pigs that were fed a diet based on SBM compared to the animals that were offered a diet containing RSM and legume plants. That result could be explained by the fact that pigs on the SBM diet had higher body weights at slaughter. On the contrary, Zaworska et al. [7] recorded a reduced back fat thickness, however, the pig body weight at slaughter was lower in their study than here. Considering the physicochemical assessment of meat, a significant difference in the acidification of muscle tissue 45 min *post mortem* was observed between the gilts and barrows. In female pigs, the pH_45_ was higher, which could be explained by a faster glycogen turnover in muscles [45]. Nevertheless, it should be emphasized that the acidification of muscle was similar for both sexes 48 h *post mortem*. The pH is directly influenced by environmental factors and pre-slaughter treatment [45]. A similar study was performed by Bocian et al. [5] on F1 hybrids (PLW × PL). Two dietary groups were applied: the control pigs were fed a diet based on SBM, whereas the experimental pigs were fed complete feed mixes with 20% pea and 37.6% yellow lupin content in the first fattening phase, and 38.5% peas plus 20% yellow lupin in the second phase. In that study, the pH measured 48 h *post mortem* was higher than here and amounted to 5.43.

In this study, the crude protein content in pork meat was found to be higher in the pigs that were fed RSM and legume plants compared to the other studies of Zralý et al. [46] and Mordenti et al. [47]. This parameter could be higher due to a simultaneous application of several legume species and RSM.

Another meat quality trait is the color. According to Szulc and Skrzypczak [48], the meat color is directly correlated with its water holding capacity; the lighter the color of the meat, the higher its water content. Hanczakowska et al. [31] assessed the meat color in fatteners that were fed diets containing yellow lupin (Mister variety). The authors recorded a lightness value of *L 51.50, whereas in our study, the meat in the experimental group had a lightness value of *L 50.60. Kuźniacką et al. [49] studied highly productive white breeds and Pulawska pigs, and they did not show differences in meat quality between the pigs that were fed diets containing legume plants (yellow lupin, pea) with RSM, and pigs fed SBM. Similarly, Chrenková et al. [50] did not find significant differences in pork meat between the pigs that were fed a diet containing peas (30%) and the fatteners that were fed a diet based on soybean meal.

## 5. Conclusions

Based on this study, it can be assumed that in DanBred pigs, feeding with feed mixes containing legume seeds, including pea, yellow lupin, and RSM, might significantly worsen the body weight gain but only in the last stage of fattening. The use of legume seeds and rapeseed meal did not worsen the meat quality of the pigs. However, pigs fed diets with an SBM substitute were characterized by statistically significantly lower mean back thickness.

## Figures and Tables

**Table 1:** Chemical composition of legume seeds (g/kg dry matter (DM)).

Component (g/kg DM)	Soybean Meal	Pea (Tarchalska)	Rapeseed Meal	Yellow Lupin (Mister)
Crude protein	463 ^1^	213	349	433
ADF	79	92	187	191
NDF	112	153	259	245
Crude fat	19	18	33	32
Starch	nd ^2^	578	nd ^3^	nd ^3^
Essential amino acid (g/16 g N)				
Arginine	7.54	12.1	6.03	15.2
Cysteine	1.35	0.89	2.61	1.78
Glycine	4.29	4.18	5.48	4.22
Histidine	2.51	2.78	2.75	3.08
Isoleucine	4.63	3.92	3.39	4.38
Leucine	7.25	7.12	7.14	8.81
Lysine	5.59	7.85	5.23	6.68
Methionine	1.17	0.52	2.05	0.51
Phenylalanine	4.82	4.51	4.28	4.47
Threonine	3.48	3.52	4.63	3.66
Valine	4.15	4.52	5.1	4.09
Alanine	4.28	4.12	4.42	3.38
Asparagine acid	11.9	12.3	7.99	11.6
Glutamine acid	18.5	15.9	15.4	25.0
Proline	5.25	3.74	6.28	3.52
Serine	5.00	4.40	4.49	5.56
Tyrosine	3.40	2.63	3.10	3.22
Antinutrients				
Total alkaloids	-	-	-	0.0011
RFO ^3^	46.62	62.85	20.5	134.2
Raffinose	7.72	8.30	3.49	10.2
Stachyose	35.1	27.10	17.1	82.8
Verbascose	3.08	27.45	-	41.2
Phytic P	4.11	1.86	7.75	4.92
Tannin	-	0.025	-	-

^1^ Each value represents the mean of four replicates. nd—not determined. ^2^ Starch wasn’t determined because the content of this compound in lupins is very low (around 1%) and the reliability of results is arguable ^3^ RTO—raffinose type oligosaccharides. NDF—neutral detergent fiber; ADF—acid detergent fiber.

**Table 2:** Chemical composition of mixed feeds for pigs of the control and experimental groups, in two feeding periods.

The Feed Ingredient (%)	Grower		Finisher	
	I—CON	II—EXP	I—CON	II—EXP
Triticale grain	81.92	76.52	87.66	83.51
Soybean meal	13.00	-	10.00	-
Rapeseed meal	-	-	-	5.00
Yellow lupin, cv. Mister	-	6.00	-	4.00
Pea, cv. Tarchalska	-	6.00	-	5.00
Brewer’s yeast	-	4.20	-	-
Rapeseed oil	2.40	2.50	-	-
Potato protein	-	2.00	-	-
Premix	0.50	0.50	0.50	0.50
Monocalcium phosphate	0.33	0.30	0.25	0,26
Fodder chalk	1.20	1.20	0.90	0.90
Fodder salt	0.30	0.23	0.22	0.23
L-lysine	0.20	0.17	0.16	0.30
DL-methionine	0.04	0.08	0.03	0.04
L- threonine	0.11	0.30	0.28	0.26
Calculated nutritional value of experimental diets (%)				
ME, MJ	12.5	12.5	12.0	12.0
Crude protein, g	144	144	135	135
Calcium, g	7.02	7.02	5.65	5.65
Ether extract, g	38.4	38.4	14.9	14.9
Crude fiber, g	28.0	30.6	27.4	34.2
Phosphorus g	4.63	4.63	4.39	4.39
Sodium, g	1.33	1.33	1.00	1.00
Lysine, g	8.77	8.77	6.6	6.6
Methionine, g	3.10	3.10	4.15	4.15
Threonine, g	6.41	6.41	4.35	4.35
Tryptophan, g	1.71	1.71	1.25	1.25
Valine, g	7.20	7.20	4.48	4.48

**Table 3:** Body weight gain at different weighing timepoints.

Group	Diet	Sex	Initial Body Weight (kg)	Grower Body Weight (kg)	Final Body Weight (kg)	Daily BWG Grower (kg)	Daily BWG Finishing Pig (kg)	Total Daily BWG (kg)
1	CON	male	27.7	61.3	118	0.961	1.206	1.102
2	CON	female	27.8	64.9	120	1.005	1.169	1.097
3	EXP	male	26.0	61.4	119	0.954	1.060	1.011
4	EXP	female	28.1	60.7	114	0.929	1.065	1.003
SEM			0.219	0.690	1.339	0.013	0.017	0.014
*p*			0.808	0.115	0.201	0.153	0.001	0.008
Main effect								
	CON		27.74	63.1	119.1 ^a^	0.9866	1.185 ^a^	1.099 ^a^
	EXP		28.17	61.0	113.6 ^b^	0.9414	1.063 ^b^	1.007 ^b^
		male	27.9	61.3	116	0.958	1.133	1.057
		female	28.0	62.8	117	0.970	1.115	1.050
*p*								
diet			0.929	0.120	0.041	0.085	0.001	0.001
sex			0.346	0.282	0.562	0.622	0.533	0.794
Diet x sex			0.825	0.114	0.799	0.144	0.434	0.954

a,b—columns marked with different letters differ significantly between groups, *p*-value < 0.05; CON = control group, EXP = experimental group, BWG = body weight gain.

**Table 4:** Some parameters of carcasses.

Group	Diet	Sex	Meatiness (%)	Carcass Length (cm)	Mean Back Thickness from Five Points (cm)
1	CON	Male	59.10	91.90	2.21
2	CON	Female	59.88	94.80	2.31
3	EXP	Male	59.72	94.10	2.00
4	EXP	Female	59.23	95.10	1.98
SEM			0.277	0.569	0.055
*p*			0.725	0.184	0.084
Main effect					
	CON		59.49	93.35	2.26 ^a^
	EXP		59.48	94.60	1.99 ^b^
		Male	59.41	93.00	2.11
		Female	59.56	94.95	2.15
*p*					
diet			0.977	0.266	0.013
sex			0.800	0.087	0.691
Diet x sex			0.269	0.397	0.570

^a,b^—columns marked with different letters differ significantly between groups, *p*-value < 0.05; CON = control group, EXP = experimental group.

**Table 5:** Physicochemical parameters of pork meat (means).

Group	Diet	Sex	pH_45_	pH_48_	Color ^1^			Water Holding Capacity (%)	Thermal Drip (%)	Drip Loss (%)
					L*	a*	b*			
1	CON	Male	6.06	4.36	51.34	6.61	5.21	37.97	17.58	5.38
2	CON	Female	6.14	4.25	51.54	6.43	5.06	38.23	18.35	5.49
3	EXP	Male	6.07	4.34	50.23	6.54	4.67	38.01	17.89	5.25
4	EXP	Female	6.23	4.44	50.98	6.74	5.04	38.53	16.35	5.52
SEM			0.018	0.074	0.549	0.202	0.204	0.509	0.401	0.206
*p*			0.001	0.855	0.853	0.962	0.829	0.979	0.340	0.970
Main effect										
	CON		6.10	4.31	51.44	6.52	5.13	38.10	17.96	5.43
	EXP		6.15	4.39	50.60	6.64	4.86	38.27	17.12	5.38
		Male	6.07 ^b^	4.35	50.79	6.57	4.94	37.99	17.73	5.31
		Female	6.19 ^a^	4.35	51.26	6.58	5.05	38.38	17.35	5.50
*p*										
diet			0.162	0.574	0.463	0.770	0.518	0.876	0.296	0.907
sex			0.001	0.977	0.678	0.985	0.793	0.704	0.632	0.662
Diet x sex			0.192	0.506	0.811	0.657	0.536	0.899	0.155	0.856

^a,b^—columns marked with different letters differ significantly between groups, *p*-value < 0.05; ^1^ L*, lightness; a*, redness; b*, yellowness; CON = control group, EXP = experimental group.

**Table 6:** The chemical composition of pork.

Group	Diet	Sex	Protein (%)	Collagen (%)	Fat (%)	Water (%)
1	CON	Male	23.66	0.70	3.03	73.01
2	CON	Female	23.67	0.70	2.74	73.35
3	EXP	Male	23.99	0.70	2.83	73.07
4	EXP	Female	23.65	0.68	2.77	73.06
SEM			0.079	0.016	0.111	0.098
*p*			0.365	0.982	0.801	0.626
Main effect						
	CON		23.67	0.70	2.89	73.18
	EXP		23.82	0.69	2.80	73.06
		Male	23.83	0.70	2.93	73.04
		Female	23.66	0.69	2.75	73.20
*p*						
diet			0.332	0.838	0.709	0.565
sex			0.302	0.884	0.444	0.419
dietxsex			0.277	0.748	0.612	0.388

CON = control group, EXP = experimental group.
